# Indications and Interpretation of Stress Radiographs in Supination External Rotation Ankle Fractures

**DOI:** 10.7759/cureus.38092

**Published:** 2023-04-25

**Authors:** Ferras Zeni, Daniel R Cavazos, José A Bouffard, Rahul Vaidya

**Affiliations:** 1 Orthopedics, Allina Health, St. Paul, USA; 2 Orthopaedic Surgery, Wayne State University School of Medicine, Detroit, USA; 3 Orthopaedic Surgery, Detroit Medical Center, Detroit, USA

**Keywords:** gravity stress, superior clear space, medial clear space, lateral malleolus fractures, ankle fractures

## Abstract

Introduction

In supination external rotation (SER) ankle fractures with an intact medial malleolus, stability hinges upon the competence of the deltoid ligament. The purpose of this study is to define the indications and establish criteria for a positive stress radiograph.

Methods

This is a prospective study of 27 isolated SER lateral malleolar fractures with a reduced ankle mortise. Pain and swelling were noted about the medial ankle, followed by an ultrasound to evaluate the integrity of the deltoid ligament. Static and stress radiographs were performed on both the fractured and contralateral ankles.

Results

Fourteen patients were normal on ultrasound examination, eight had partial tears, and five had full-thickness tears. The difference in the level of pain to palpation postero-medially between the complete tear (7 +/- 1) and the partial tear (1.3 +/- 2.4) group was significant (p < .001). The negative predictive values for medial swelling and tenderness were 93% and 100%, respectively. Sensitivity and specificity for medial clear space on stress radiograph (fracture (fx)) > 5.0 mm were both 100% while a 2.5 mm or greater change to the contralateral side yielded a sensitivity of 100% and specificity of 95%.

Conclusion

The lack of significant medial pain, as well as swelling, implies the absence of a complete ligament tear and eliminates the need for stress examination. Conversely, the presence of medial signs of injury is suggestive, but not pathognomonic for a complete deltoid tear. Medial clear space (MCS) variability prompts to recommend a minimum of 2.5 mm on stress radiographs compared to the contralateral side as indirect evidence for a complete tear of the deltoid ligament.

## Introduction

Historically, treatment of isolated lateral malleolus fractures has depended upon the clinician’s assessment of overall ankle stability and the likelihood of returning to a normal gait pattern. Numerous studies have demonstrated that supination external rotation (SER) II injuries can be treated non-operatively with results equivalent to surgery with lateral malleolus displacement up to 5 mm [1-6].

Ankle stress radiographs in the setting of an isolated lateral malleolar fracture have recently gained popularity in assessing ankle stability [7-9]. However, stress views date back to Lauge-Hansen who both advocated the use of external or lateral rotation stress radiographs over fifty years ago [10]. Kleiger used stress radiographs as early as 1944 and even devised an apparatus in an attempt to standardize stress radiographs [11]. In 1969, Phillips et al. performed a prospective study of 31 patients with oblique external rotation fractures who all underwent external rotation stress radiographs under general anesthesia [5]. They found that four of thirty-one patients were unstable and required closed reduction [5].

Sequential sectioning experiments of the deltoid have demonstrated no contribution of the superficial deltoid alone to resisting lateral talar subluxation in contradistinction to isolated deep deltoid division [12-14]. Thus, treatment of these fractures depends upon the integrity of the deep deltoid ligament [15]. This concept has been validated by several clinical studies [1,4,16]. Therefore, in nondisplaced SER ankle fractures, one must make an accurate assessment of the integrity of the deltoid ligament to determine ankle stability [16-17]. Intuitively, one would assume that the lack of medial tenderness would eliminate the possibility of a deltoid injury, albeit partial or complete. However, many studies have suggested otherwise [16-17].

Stress radiographs can be performed to detect any abnormal medial clear space widening [18-19]. Unfortunately, the interpretation of these radiographs can be challenging since the criteria for instability on stress radiographs are debated [18-21]. Tornetta was the first to recommend using medial clear space on a stressed radiograph (MCSS) > 4 mm and MCSS - SCS (superior clear space) greater than 1 mm as a cutoff for instability [19]. Using these criteria, McConnell et al. found that medial tenderness sensitivity and specificity for a complete tear were 28% and 67%, respectively [7]. However, these criteria are based on medial clear space changes in a cadaveric experiment as the result of an axial load and not an external rotation force [22]. Thus, one must question the validity of these criteria.

Many studies have since followed advocating the use of MCSS > 4 mm as a cutoff for instability and have found no relation between physical exam and stress radiograph results [7-8,22]. There is no study predating Tornetta’s study that advocates 4 mm as a cutoff for dynamic instability on stress radiographs [19]. Furthermore, no study exists that definitively correlates the status of the deltoid ligament objectively (arthroscopically, operative exploration, MRI, ultrasound, etc.) with stress radiographs.

Harper, Michelson, and Baird, all advocated the use of 5 mm as a cutoff for instability on initial unstressed radiographs which is caused by a “substantial injury” of the deltoid ligament [15,22,23]. Alternatively, Baird believed that a medial clear space greater than superior clear space on unstressed radiographs, as initially suggested by Charnley, was indicative of lateral talar subluxation [23-24]. Phillips et al. used 4 mm as a cutoff for what was deemed an acceptable reduction after a displaced ankle fracture [5]. 

An alternative to the manual stress exam is the gravity stress test as described by Michelson [22]. According to his cadaveric study, a 2 mm or greater change in the medial clear space was considered a positive gravity stress test [22]. Two recent prospective studies have used the gravity stress test to assess ankle stability [25-26]. These studies are potentially problematic since Tornetta’s criteria for instability are utilized and not the original criteria as described by Michelson [22].

Thus, we devised a study to first accurately assess the integrity of the deltoid ligament utilizing ultrasound and then correlate these findings with physical exam and stress radiographs of both the fractured and normal side. We hypothesized that this would 1) validate the role of the deep deltoid in maintaining ankle stability in SER fractures, 2) verify the importance of a detailed physical exam in predicting deltoid injury, 3) establish indications for an ankle stress test, and 4) help define criteria for a positive stress test.

## Materials and methods

Patient population

This was an IRB-approved (Outcomes of Fractures: 020717MP4E) prospective study that enrolled 27 consecutive patients with supination-external rotation injuries according to Lauge-Hansen over a four-month period. Exclusion criteria were 1) medial malleolar fracture 2) medial clear space widening > 4 mm on initial injury films, 3) evidence of peripheral neuropathy, 4) previous ipsilateral ankle fracture, 5) skeletal immaturity, and 6) evidence of syndesmotic injury. 

Clinical evaluation

An initial clinical encounter form was completed for each patient. Patients were formally evaluated in the clinic after their initial ER visit and the post-injury day was noted. A single author evaluated all patients. The presence of swelling, pain, and tenderness to palpation was noted. Swelling was graded as none/minimal, mild, moderate, and severe (defined as fracture blisters). Patients who graded their pain on palpation as mild or greater were considered to be clinically important. Each patient was also asked to rate their tenderness to palpation at four locations medially (medial malleolus, anterior, inferior, and posterior to the medial malleolus) and four locations laterally (lateral malleolus, anterior, inferior, and posterior to the lateral malleolus) according to the visual analog score (0 to 10). The four values for each side were then averaged yielding an average medial and lateral visual analog score.

Radiographic evaluation

**Figure 1:**
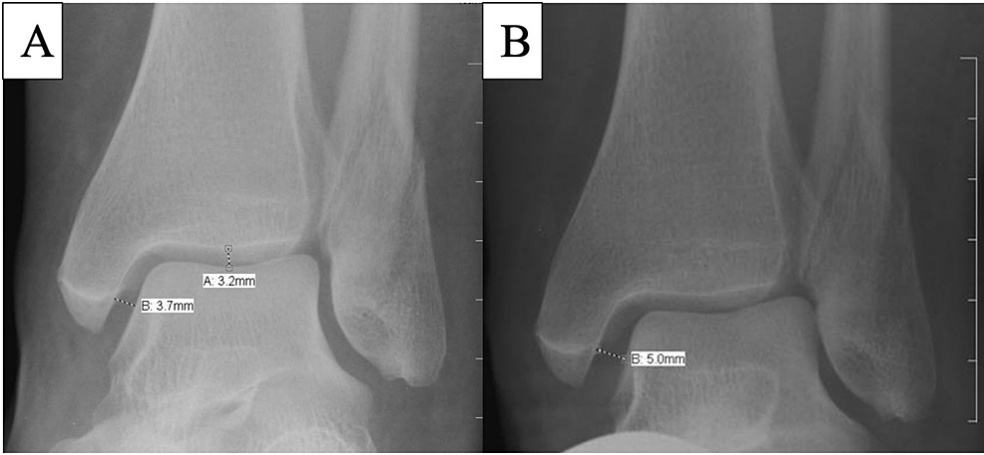
Figure 1A showing a mortise view with measurement A for SCS measuring 3.2 mm (A) and measurement B for MCS of 3.7 mm (B). Figure 1B showing the stress radiograph with MCS widening of 5.0 mm (B). MCS: Medial clear space; SCS: Superior clear space

Radiographs were obtained utilizing the Kodak (Rochester, USA) CR 900 imaging system. Static radiographic evaluation included an ankle trauma series (anteroposterior, mortise, and lateral) of the fractured ankle and a mortise view of the contralateral ankle. An external rotation stress radiograph (mortise) of both the fractured and the uninjured side were performed (Figure 1A-1B). Unsatisfactory static and dynamic mortise radiographs were repeated.

The above measurements were done by a single author using the Hospital Picture Archive and Communications System (PACS) (Stentor, San Francisco, USA). The pixel resolution of the images was 0.12 mm, making this the limiting accuracy in the measurements. Each parameter was measured to the tenth of a millimeter on the mortise view. The superior clear space (SCS) was measured at its widest point in the center of the tibiotalar joint. The MCS was measured tangentially from the mid portion of the articular surface of the medial malleolus, approximating the distal extent of the posterior colliculus, to the medial border of the talus. This was used since it is a readily identifiable landmark. Radiographs could be substantially magnified on the computer display to better identify pertinent landmarks.

Ultrasonographic evaluation

Ultrasound examination of the deltoid ligament was performed by one of two musculoskeletal radiologists. The status of each component of the deltoid ligament was noted similar to Rasmussen et al.’s description which is based on the origins and insertions of the ligaments [26]. This is similar to Pankovich and Shivaram’s description superficially and includes the tibionavicular and the tibiocalcaneal ligaments [27]. The tibiotalar component which is deep consists of the anterior and posterior tibiotalar ligament originating from the anterior and posterior colliculus, respectively. Patients were classified as intact, partial tear, and complete tear. Ultrasonographic findings for an intact deltoid ligament included ligaments with the normal linear ligamentous pattern without change in morphology or signal alteration. The partial tear group included findings of irregular fibers with a change from the normal linear morphology with interstitial edema. The complete tear group had complete disruption of the normal linear pattern of ligamentous fibers. Patients with a completely torn tibionavicular, tibiocalcaneal, anterior tibiotalar and a partially torn posterior tibiotalar ligaments were considered a part of the complete tear group. 

## Results

Demographics

Twenty-seven consecutive patients were enrolled. Fifteen were female (56%) and twelve were male (44%). The average age was 51 (range 15-92) years. None of the fractures was the result of a high-energy injury.

Ultrasound evaluation

**Figure 2:**
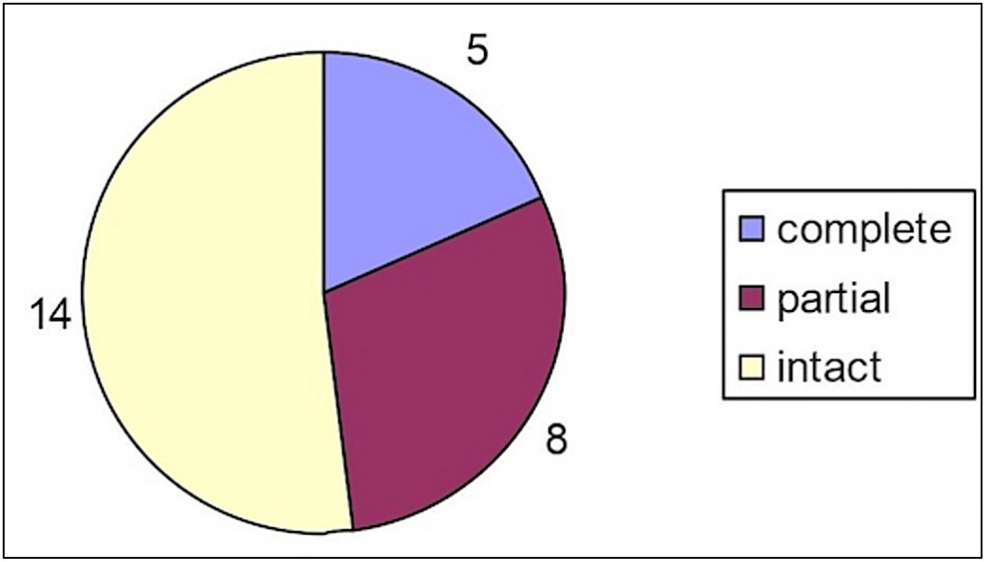
Ultrasound measurements for deltoid evaluation (partial, complete, or intact).

A total of 14 patients had no evidence of a deltoid ligament tear (52%), eight had a partial tear (30%), and five had a complete tear (18%) including two patients with a partial tear of the posterior tibiotalar ligament as described earlier (Figure 2).

Medial swelling and pain

**Figure 3:**
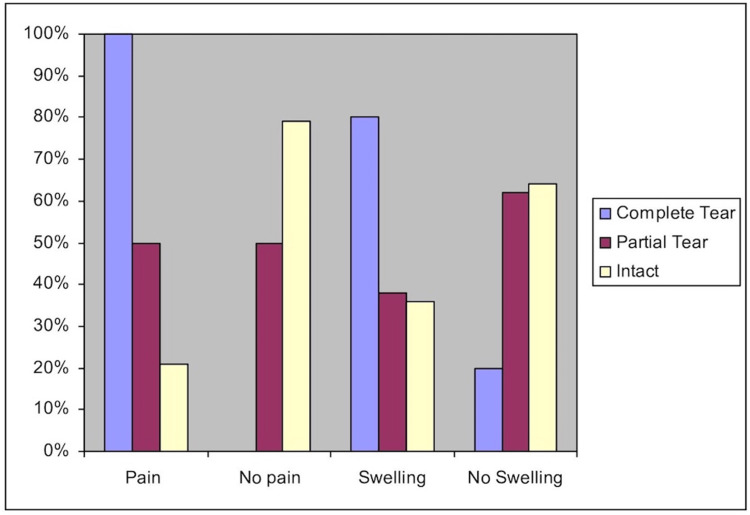
Bar graph comparing pain and swelling to severity of tear.

The patients had their formal clinical evaluation at the time of their initial follow-up in clinic. On average, patients were evaluated on post-injury day four (range 0-7) days. The presence of pain on physical exam was seen in 100% of complete deltoid ligament tears as seen in Figure 3. However, partial tears and intact deltoid ligament complexes had a variation in the presence of pain. Swelling was present in 80% of complete tears, but 20% (one out of five patients) had no swelling and a complete tear. In addition, 60% of partial tears had no swelling at all.

The positive predictive value, negative predictive value, sensitivity, and specificity were obtained for the presence of medial swelling and pain to the prediction of a complete deltoid injury. Most notably, negative predictive value for medial swelling and tenderness was 93% and 100%, respectively, for a deltoid injury (Table 1).

**Table 1:** Physical exam parameters of medial swelling and pain and their prediction for complete deltoid ligament injury compared to ultrasound.

Physical Exam Parameter	Positive Predictive Value	Negative Predictive Value	Sensitivity	Specificity
Medial swelling	33%	93%	80%	64%
Medial pain	42%	100%	100%	68%

Visual analog score

**Figure 4:**
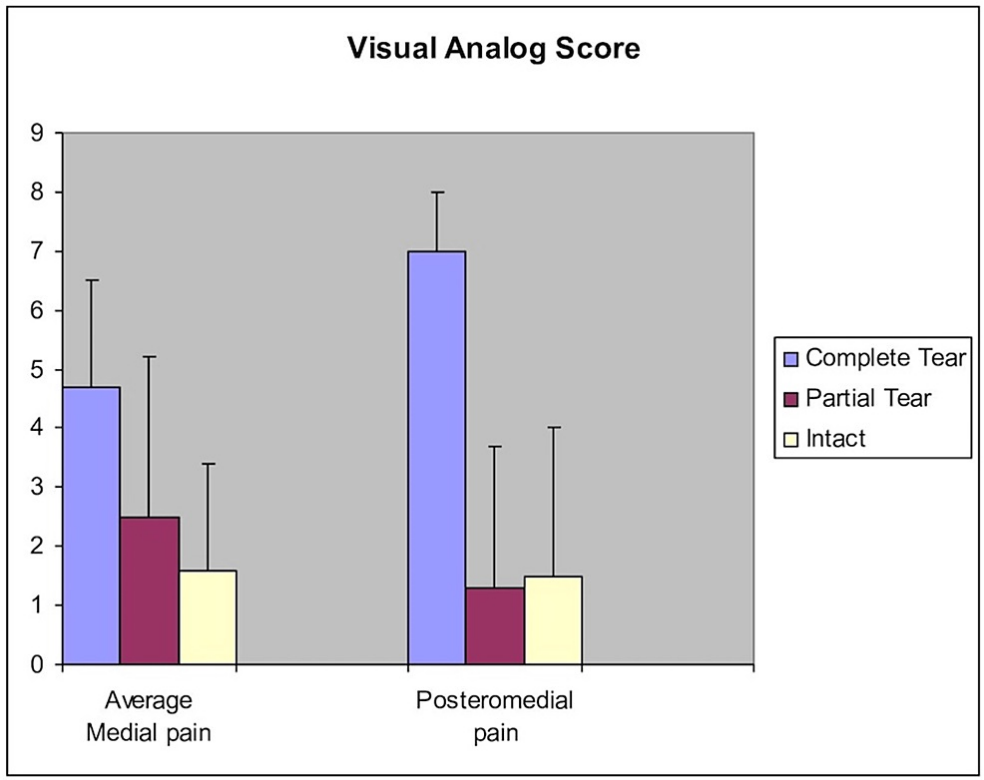
Medial pain versus posteromedial pain bar graph and severity of tear.

Patients with a complete tear, on average, had greater medial pain than the intact group (p = 0.010), but not the partial tear group (p = 0.079) or the partial versus intact group (p = 0.361) (Table 2). We also looked at posteromedial pain since this seemed to correlate most strongly with a complete tear. Patients with a complete tear had statistically more posteromedial pain than both the partial tear and intact groups (p < 0.001) (Figure 4).

**Table 2:** P-values for severity of tear versus medial or posteromedial pain

Physical Exam Parameter	Complete vs. Intact	Complete vs. Partial	Partial vs. Intact
Average medial pain	p = .010	p = .079	p = .361
Posteromedial Pain	p < .001	p < .001	p = .807

Radiographic examination

All 27 patients had bilateral stress mortise radiographs. A single author performed 24/27 stress radiographs on the fractured ankles. All unsatisfactory stress views were repeated. 

Static radiographic values

**Figure 5:**
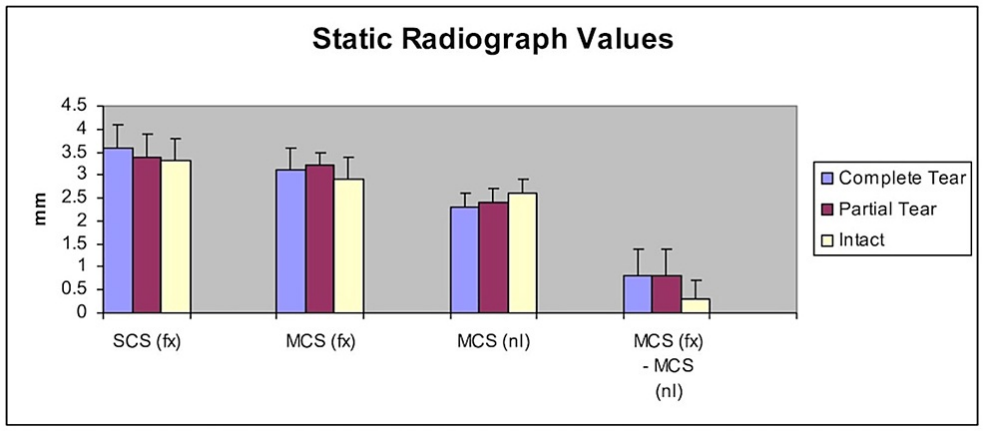
Static radiograph value parameters comparing complete tear, partial tear, and intact deltoid ligaments. MCS (fx): Medial clear space (fracture side); MCS (nl): Medial clear space (normal or contralateral extremity); SCS (fx): Superior clear space (fracture side)

Superior clear space (fracture) “SCS (fx)”, medial clear space (fracture) “MCS (fx)”, medial clear space (normal or contralateral ankle) “MCS (nl)”, and MCS (fx) - MCS (nl) were measured on static radiographs. There was no significant difference between the various groups with regards to SCS (fx), MCS (fx), and MCS (nl) when comparing a complete tear, partial tear, and intact deltoid ligaments (Figure 5).

The MCS (fx) - MCS (nl) parameter was statistically significant in the complete tear group (0.8 +/- .6 mm) and partial tear group (0.8 +/- .4 mm) when compared to the intact group (0.3 +/- .4 mm) (Table 3).

**Table 3:** Statistical values for the dynamic stress radiograph parameters for the complete tear, partial tear, and intact groups. MCS (fx): Medial clear space (fracture side); MCSS (fx): Medial clear space on a stressed radiograph (fracture side); MCS (nl): Medial clear space (normal or contralateral extremity); SCS (fx): Superior clear space (fracture side)

Radiographic Parameter	Complete vs. Intact	Complete vs. Partial	Partial vs. Intact
MCS (fx) - MCS (nl)	p = .048	p = .851	p = .013
MCSS (fx)	p < .001	p < .001	p = .13
MCSS (fx) - MCS (nl)	p < .001	p < .001	p = .055
MCSS (fx) - MCS(fx)	p < .001	p < .001	p = .461
MCSS (fx) - SCS(fx)	p < .001	p = .003	p = .229

Dynamic radiographic values

Medial clear space on a stressed radiograph in the fractured (fx) ankle (MCSS (fx)) is a dynamic measurement to assess ankle stability. The mean MCSS (fx) value for the complete tear group was 6.6 +/- 1.7 mm (5.4-9.4 mm), 4.5 +/- .4 mm (4.0-5.0 mm) for the partial tear group, and 3.9 +/- .5 mm (2.9-4.7 mm) in the no tear group. Differences amongst the groups were significant except for the superficial versus intact group (Table 3).

**Figure 6:**
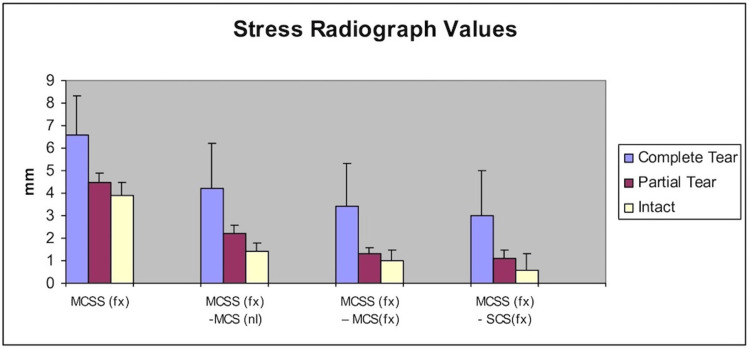
Dynamic stress radiograph parameters for the complete tear, partial tear, and intact groups MCS (fx): Medial clear space (fracture side); MCSS (fx): Medial clear space on a stressed radiograph (fracture side); MCS (nl): Medial clear space (normal or contralateral extremity); SCS (fx): Superior clear space (fracture side)

MCSS (fx) - MCS (nl), MCSS (fx) - MCS (fx), and MCSS (fx) - SCS (fx) parameters were also obtained with numerical values shown in Figure 6 and statistical values in Table 3. All parameters were significant (p < 0.05), for the complete versus intact and partial groups, but were not significant for the partial versus intact group. However, the MCSS (fx) - MCS (nl) partial versus intact group was approaching significance (p = 0.055).

Lastly, common radiographic parameters were assessed for their statistical accuracy of deltoid ligament stability compared to ultrasound as listed in Table 4.

**Table 4:** Common radiographic parameters statistical values to assess for ankle stability. MCS (fx): Medial clear space (fracture side); MCSS (fx): Medial clear space on a stressed radiograph (fracture side); MCS (nl): Medial clear space (normal or contralateral extremity); SCS (fx): Superior clear space (fracture side)

Radiographic Parameter	Positive Predictive Value	Negative Predictive Value	Sensitivity	Specificity
MCS > SCS	40%	86%	40%	86%
MCSS (fx) > 5	100%	100%	100%	100%
MCSS (fx) ≥ 5	71%	100%	100%	91%
MCSS (fx) ≥ 4	24%	100%	100%	27%
MCSS (fx) > 4 mm + MCSS (fx) - SCS (fx) ≥ 1.0	50%	100%	100%	77%
MCSS (fx) - MCS (nl) ≥ 3.0	100%	92%	60%	100%
MCSS (fx) - MCS (nl) ≥ 2.5	83%	100%	100%	95%
MCSS (fx) - MCS (fx) ≥ 2	100%	100%	100%	100%
MCSS (fx) - SCS (fx) ≥ 1.5	57%	95%	80%	86%

## Discussion

The definitive interpretation of stress radiographs is a matter of debate. Various radiologic criteria have been used to assess stability (Table 5) [7,10,19]. Each measurement has its inherent problems. Relying on an absolute measurement of medial clear space on a stressed radiograph (MCSS) does not take into account the variability of the normal distribution of medial clear space (1.8-3.6 mm in this study and 1.3-4.3 mm in another study) [5]. Relying on the MCS (fx) or SCS (fx) space as a reflection of the normal MCS is not accurate since according to this study, they are on average 0.5 mm (range -0.1 - 1.6 mm) and 1.0 mm (range 0.2 - 1.7 mm) greater than MCS (nl), respectively. Although the magnitude of this range is small, significant measurement changes occur on the order of sub-millimeters. In theory, it is the change between the ankle’s native MCS and MCSS (fx) that should provide the most accurate representation of talar subluxation and most importantly, instability.

**Table 5:** Radiographic criteria in literature used to assess stability of the medial ankle [7,10,19]. MCSS: Medial clear space on a stressed radiograph; MCS: Medial clear space; SCS: Superior clear space; LCS: Lateral clear space

Radiographic Criteria for Instability
MCS > 4 mm
MCS > SCS
MCSS > 5 mm
MCSS > 4.5 mm
MCSS ≥ 4 mm
MCSS > 4 mm and MCSS - SCS > 1
MCSS - MCS > 2-3 mm
MCS - LCS > 3

Various cadaveric experiments have investigated changes in lateral talar subluxation as a result of sequential ligamentous sectioning and lateral malleolar osteotomy. Harper demonstrated that with the lateral malleolus intact, no talar shift or tilt was possible [14]. He also demonstrated increasing amounts of instability with anterior inferior tibiofibular ligament (AITFL)sectioning and lateral malleolar osteotomy (SER II) yielding lateral talar subluxation of 1.2 mm on average (range 1-1.4 mm) with manual pressure which increased to 2.3 mm (range 2-3 mm) with posterior inferior tibiofibular ligament (PITFL)sectioning (SER III) [12-15]. With deep deltoid sectioning, lateral talar subluxation increased to 3.8 mm (3-4.5 mm) [12-15]. The summation of these studies is that the intact deltoid consistently allows up to three mm of lateral talar subluxation with no subsequent increase until the deep deltoid is cut. It is also important to note that a cutoff of 5 mm on stress radiographs was also indicative for an SER IV lesion [15,22-23].

However, the ability to use ultrasound to image the deltoid ligament allows us to classify the extent of injury and then correlate these results with our stress radiographs and physical exam. The high sensitivity and specificity of ultrasound in assessing deltoid ligament injury combined with our ability to accurately measure radiographs using the PACS system allowed us to validate stress examination as a viable diagnostic tool.

A total of five patients had a complete tear of the deltoid ligament. The diagnosis of instability based on stress radiographs was straightforward in this group of patients. All five patients had an MCSS (fx) > 5 mm. Both sensitivity and specificity were 100% for MCSS > 5 mm. However, as explained above, relying on this number as an absolute cutoff for MCSS (fx) could be problematic. A false negative or positive could result in patients with SER IV fractures and a small MCS (nl) or patients with SER II fractures and a large MCS (nl), respectively. With a larger patient population, we would have been more likely to observe this possibility.

When looking at changes in medial clear space on stress radiographs, both MCSS (fx) - MCS (nl) > 2.5 mm and MCSS (fx) - MCS (fx) > 2 mm yielded a high sensitivity and specificity as well. The reason for the difference between the two cutoffs is because of a consistently observed increase in MCS (fx) compared to MCS (nl) (average). However, since the increase in MCS (fx) was variable, one should also interpret any change on stress radiographs relative to MCS (fx) with caution.

The importance of evaluating instability based on a change in the medial clear space relative to the contralateral medial clear space can be seen when comparing all three of the mentioned parameters graphically. The distribution of individual values based on the extent of deltoid ligament damage is best differentiated in the MCSS (fx) - MCS (nl) graph.

In the complete tear group, the two patients with partial tears of the posterior tibiotalar ligament had the smallest MCSS (fx) - MCS (nl) values (2.5 and 2.8 mm). Because of the high-grade nature of their tear, they were thought to have potentially incompetent ligaments. Thus, they were included in the complete tear group and underwent operative fixation. However, the presence of some intact fibers led to less than a 3 mm difference with stress exam as would have been predicted by cadaveric sectioning experiments. It is uncertain whether these patients could have been treated successfully with immediate weight bearing given a portion of the posterior tibiotalar was intact. The remaining three patients in this group all had an MCSS (fx) - MCS (nl) > 3 mm (range 3.6-7.3 mm).

Two of the patients in the complete tear group did not undergo operative fixation, one patient was not medically stable, and the other patient elected cast immobilization. They were treated in a non-weight-bearing cast for six weeks and progressed to full weight-bearing healing uneventfully by ten weeks (MCS (healed) = 2.7 mm and 1.9 mm).

In the partial tear group, there was a trend toward increasing instability on stress radiographs when compared to the intact group. MCSS(fx) - MCS (nl) ranged from 1.8-2.9 mm (2.3 +/- .4 mm) for the partial tear group versus 0.6-1.8 mm (1.4 +/- .4 mm) for the no tear group. This difference between these two groups approached statistical significance (p = 0.055).

Three patients in the partial group had an MCSS (fx) approaching 5 mm. These three patients served as a source of apprehension, unlike the remainder of the patients with partial tears. Because these three patients did not have any signs of posterior tibiotalar involvement on ultrasound, surgery was not recommended. They were placed into a non-weight-bearing cast and allowed progressive weight-bearing starting at 4-6 weeks. One patient sought a second opinion and underwent operative fixation at an outside institution. The remaining two patients also healed uneventfully (MCS (healed) = 3.8 mm and 2.3 mm).

Using MCSS > 4 mm as a cutoff for instability in our patient population, as advocated by Egol et al., yielded a sensitivity of 100%, specificity of 27%, and a positive predictive value of 24% with regards to a complete tear of the deltoid [17]. Egol et al. questioned whether a positive stress radiograph in the absence of medial signs is an indication for surgery since all patients in this group who were treated nonoperatively either had good or excellent results [17]. They concluded that “the indication for surgery should not be based on the absolute value of the widening of the medial clear space noted on the stress radiograph”. Our study confirms that suspicion and proves that their criteria are not stringent enough and would lead to many false positives. Adding to this the condition that MCSS fx - SCS (fx) > 1 mm increases specificity to 77% but still yields a low positive predictive value of 50%. 

Unlike previous studies, we found physical exam to be helpful in evaluating patients for potential deltoid ligament damage [16-17,28]. Physical exam was most helpful in excluding the possibility of a complete tear. All patients who lacked pain and 93% who lacked swelling did not have a complete tear. Overall, the sensitivity of medial pain was 100% for the complete tear group and specificity was 67% (Table 1). Because of the low specificity, we do agree with previous studies that medial tenderness in of itself cannot be used as a sole indicator for deltoid ligament rupture.

As a group, patients with a complete tear graded their pain higher than the partial tear/intact group (p=.010). Patients in the complete tear group also had a higher average visual analog score (VAS) medially than the intact group, but not the partial tear group. The amount of posteromedial pain helped to further distinguish patients with complete tears from the other two groups. The presence of swelling was not statistically significant when comparing the complete tear group to the superficial/intact group (Fisher exact test, p=0.139). However, the negative predictive value for no swelling was 93% (partial tear/intact ligament). Furthermore, 80% of patients with a complete tear had swelling while this was only true in 38% and 36% of the patients in the partial tear and intact groups, respectively. This suggests a trend toward increasing swelling in the complete tear group. Thus, swelling, and more importantly, pain, can help stratify risk in patients with potential instability since all patients with a complete tear did have pain.

Thus, we believe the reason why previous studies have uniformly suggested no association of physical exam with the status of the deltoid ligament is because of the low specificity of their criteria as demonstrated by this study. As mentioned previously, these criteria as initially suggested by Tornetta are based on a cadaveric experiment utilizing an axial and not rotational load to assess ankle stability [19,22]. Studies utilizing the gravity stress test also utilize these criteria and not the criteria as originally described by Michelson [18,21,22,25,26].

Our data suggests that differentiating patients with a partial tear from those with an intact ligament is difficult based on physical exam. Fifty percent of patients with a partial tear had no pain and 62% had no swelling. This group was more similar to the normal group with regard to pain and swelling than the complete tear group. It is interesting to note that four patients with a partial deltoid tear did not have medial pain. The same could not be said of any patient with a complete tear.

One of the limitations of our study is the relatively small number of patients enrolled (27). Despite our small numbers, we find similar results if we are to analyze our data using criteria advocated by other studies. Another possible limitation of this study is that only a small subset of patients was evaluated by post-injury day one (2 of 27). However, we believe our study protocol which allowed for patient evaluation in the outpatient setting by post-injury day seven is more applicable to actual clinical practice. It is possible that this relative delay could lead to more generalized swelling making it a less reliable sign of actual injury.

Stress radiographs can also be problematic. If a perfect mortise view is not obtained with the ankle dorsiflexed, relevant landmarks are difficult to identify. Thus, repeat stress radiographs were performed on several patients. We did standardize our measurement by always measuring at the level of the distal extent of the posterior colliculus. Our study also had the advantage that 24 of 27 fractured ankles were stressed by a single author.

The lack of a rigid treatment protocol is also problematic. All patients with an intact deltoid (14) and the majority of patients with a partial tear (5/8) were allowed early weight bearing and healed without incident. The remaining three patients were treated nonoperatively in a non-weight-bearing cast since their MCSS (fx) approached 5 mm. Only after analyzing our data did the importance of comparing the stress radiograph to the normal side emerge. Two of these patients had changes on stress radiographs less than 2.5 mm while the third had a 2.9 mm increase. Based on our criteria for a positive stress radiograph, this third patient would be false positive according to the ultrasound. In addition, no prior calculation was performed, which serves as a weakness in the design of this prospective study.

It is possible that the two patients with partial tears of the posterior tibiotalar ligament could have been treated successfully nonoperatively. The decision to classify these patients as complete tears may be controversial. However, they did demonstrate a large increase in medial clear space on stress radiographs (2.5 mm and 2.8 mm). This would explain why we would suggest a minimum increase of 2.5 mm in the medial clear space on stress radiographs compared to the normal side. Future studies could be conducted to evaluate the efficacy of using 3 mm as a cutoff for instability. 

## Conclusions

Physical exam was helpful in excluding a complete tear of the deltoid with negative predictive values of 100% and 93% for pain and swelling, respectively. Thus, we recommend immediate weight bearing as tolerated in patients without medial pain without antecedent stress examination combined with close follow-up. We would reserve stress examination for patients with medial pain eliminating the need for such an exam in 55% of patients while not missing any complete tears.

When obtaining stress radiographs, we would also recommend obtaining a static radiograph of the contralateral since neither MCS (fx) nor SCS (fx) accurately approximates MCS (nl). A minimum increase in the medial clear space of 2.5 mm on stress radiographs compared to MCS (nl) should be considered a positive stress test. Using MCSS (fx) > 4 mm, even with the condition that it should be 1 mm greater than the SCS, leads to an unacceptably high number of false positives.

We did individualize treatment within the partial tear group allowing the majority of patients (5/8) immediate weight bearing which did not lead to subsequent medial clear space widening. Given a shared intact posterior tibiotalar ligament in all patients in the partial tear group, we believe that immediate weight bearing is warranted and justified in all patients who do not meet the abovementioned criteria for instability.
